# The Effect of Korean Red Ginseng on Sexual Function in Premenopausal Women: Placebo-Controlled, Double-Blind, Crossover Clinical Trial

**DOI:** 10.1155/2015/913158

**Published:** 2015-12-22

**Authors:** Ho Seok Chung, Insang Hwang, Kyung Jin Oh, Mi Na Lee, Kwangsung Park

**Affiliations:** Department of Urology, Chonnam National University, 671 Jebongno, Donggu, Gwangju 61469, Republic of Korea

## Abstract

This study investigated whether Korean red ginseng (KRG) extracts could improve sexual function in premenopausal women. Forty-one premenopausal women participated in this placebo-controlled, double-blind, and crossover clinical study with administration of either three ginseng capsules (1 g per capsule) or placebo daily. After 8 weeks of medication of KRG or placebo, medication was changed for the subjects to placebo or KRG after 2 weeks of washout period. The efficacy of KRG extracts was measured by using Female Sexual Function Index (FSFI).* Results*. Twenty-three women completed the study. Total FSFI scores increased after KRG treatment (from 20.13 ± 2.87 to 23.98 ± 4.10, *p* = 0.015) and placebo treatment (from 20.06 ± 2.64 to 23.78 ± 3.28, *p* = 0.003). However, this change was not significantly different between the two groups (*p* = 0.702). KRG treatment significantly improved sexual desire, arousal, orgasm, and satisfaction domains; however, there was no treatment effect compared with placebo. There was a case of gastric discomfort after taking KRG extracts. Oral administration of KRG extracts improved sexual function in premenopausal women; however, there were no statistical significant changes compared to placebo. It implies that KRG extracts have a substantial placebo effect in premenopausal women with sexual dysfunction.

## 1. Introduction

Female sexual dysfunction (FSD) is prevalent problem which affects approximately 40% of women [1]. FSD is a multifactorial phenomenon which is composed of medical and psychosocial aspects [2, 3]. While male sexual dysfunctions are often treated with pharmacological agents, FSD still needs further research and clinical trials to find efficacious therapeutic options [4–9].

Korean red ginseng (KRG) (*Panax ginseng* Meyer) is an herb of the family Araliaceae, which is a slow growing perennial plant with fleshy roots [10]. It grows in cooler climate regions of the Northern Hemisphere and is used as a traditional complementary and alternative medicine [11]. It contains triterpenes ginseng saponins called ginsenosides, which are responsible for its various pharmacological effectiveness, such as anti-inflammatory activity, effects on pulmonary function, and improvement of cognition in Alzheimer's disease and sexual function [12–17].

In our previous study of menopausal women, we reported that KRG improved sexual arousal [12]. In animal studies, we found that KRG has a relaxing effect on clitoral corpus cavernosal smooth muscle and vaginal smooth muscle in rabbit model [18]. Also, we found that KRG may have estrogenic effects on female rats [19]. From these results, we hypothesized that KRG may improve sexual function of premenopausal women with FSD. The aim of this study was to determine the effects of KRG on sexual function in premenopausal women.

## 2. Materials and Methods

The study protocol was reviewed and approved by an institutional review board (CRE 11156-7) of Chonnam National University Hospital.

### 2.1. Subjects

The subjects of study were premenopausal women with female sexual dysfunction in a community-based population from September 2011 to May 2012. We enrolled premenopausal women who had all of the following inclusion criteria: (1) Female Sexual Function Index (FSFI) questionnaire score less than 26.5 at the initial evaluation; (2) participants being required to have sexual intercourse interaction at least 4 times a month with a steady sexual partner; (3) women aged between 31 and 51 years with regular menstruation at least for 1 year; and (4) women who decided to participate in this study with their own will. All of the participants were provided information related to this study with written informed consent. They were also informed that they could quit the participation at any time during the study.

Exclusion criteria were as follows: (1) coronary artery disease or other heart problems such as significant arrhythmia; (2) uncontrolled diabetes mellitus; (3) uncontrolled hypertension; (4) blood pressure lower than 90/50 mmHg; (5) a history of radical hysterectomy; (6) anatomical deformity of external genitalia; (7) a history of cerebrovascular disease or central nervous system disease; (8) a history of spinal cord injury or related surgery; (9) abnormal level of serum liver enzymes; (10) serum creatinine >2.5 mg/dL; (11) intake of medicine such as amlodipine, valerian, and lorazepam (which are known to have interactions with ginseng); (12) a history of hormonal treatment for sexual dysfunction during the past two weeks; (13) a history of chemotherapy or pelvic radiation treatment; (14) sexual dysfunction which was caused by psychogenic problem; and (15) sexual dysfunction which was caused by low level of testosterone (less than 0.66 pg/mL).

### 2.2. Study Design

The study was a randomized, double-blind, and placebo-controlled crossover study. In each participant, total protocol period was 20 weeks. The first two weeks was for collecting baseline data. During the following 8 weeks, double-blind, placebo-controlled crossover study was done with randomly allocated treatment arms. Two weeks of washout period was scheduled in between them.

At initial visit (visit 1), screening was done. Medical interview, FSFI, physical examination and baseline vital sign were checked. The laboratory study including serum hormonal level (total testosterone, free testosterone, and estradiol) and urine pregnancy test was conducted. After first visit, any participants who met exclusion criteria were excluded. For all of enrolled patients, written informed consents were obtained.

At second visit (visit 2), baseline data was collected and treatment arms were started. The subjects were divided into two groups, randomly. This study was double blinded that all subjects, investigators, pharmacists, and study staff did not know to which group the subjects were enrolled. One group was asked to take six capsules of KRG daily for 8 weeks. The other group was asked to take placebo capsules with the same periods. After 8 weeks of taking medication, 2 weeks of washout period without any medication was followed. After washout period, groups were switched to the other group medication (crossover) for 8 weeks again. Detailed information for KRG and placebo capsule will be described later.

Before the beginning of each treatment (visits 2 and 5), baseline data including sexual function and hormonal levels were measured. Detailed information for sexual function measurement will be described later. Every 4 weeks during medication, the subjects were required to visit the clinic for assessment of their condition and any adverse event (visits 3, 4, 6, and 7). Sexual function and hormonal level measurement were done repeatedly at each end of the treatment arm (visits 1, 4, and 7). Due to the fact that the study was done as crossover design, all patients served as their own controls. A well-trained female staff conducts the interview with subjects. Interview for sexual function measurement and adverse events was conducted by the same female interviewer.

### 2.3. KRG and Placebo Capsules

KRG capsules and placebo capsules were provided by the Korean Ginseng Corporation (Daejeon, Korea). A KRG capsule contains 500 mg of dried ginseng powder from the root of six-year-old* Panax ginseng* C.A. Meyer. The capsule was made of hydroxypropyl methylcellulose, pectin, purified water, sucrose fatty acid ester, glycerin, calcium gluconate, and glacial acetic acid. KRG contained the following active compounds: -Rb1, 5.61 mg/g; -Rb2, 2.03 mg/g; -Rc, 2.20 mg/g; -Rd, 0.39 mg/g; -Re, 1.88 mg/g; -Rf, 0.89 mg/g; -Rg1, 3.06 mg/g; -Rg2(s), 0.15 mg/g; -Rg3(r), 0.08 mg/g; -Rg3(s), 0.17 mg/g; -Rh1, 0.30 mg/g; and other minor ginsenosides. A placebo capsule contains starch to mimic KRG capsule. The flavor and the appearance of two capsules were identical.

### 2.4. Assessment of Female Sexual Function

For evaluation of the female sexual function, FSFI was used [20]. We used the Korean version of FSFI [21]. This questionnaire consists of 19 items in 6 domains: desire (two items), arousal (four items), vaginal lubrication (four items), orgasm (three items), global satisfaction (three items), and pain (three items). The items for sexual desire and satisfaction were measured using a 5-point scale while the rest of the questions were measured with a 6-point scale. The lowest point was 4 and the highest point was 95; thus, lower scores reflected more severe disorder in terms of sexual function.

The FSFI was measured at the beginning and at the end of each medication (visits 2, 4, 5, and 7). For the assessment of secondary efficacy, we evaluated the total and each score of the 6 domains of the FSFI.

### 2.5. Analysis

At the end of the 8 weeks of each treatment arm, we compared all the variables between the ginseng group and placebo group. Chi-square test, *t*-test (crossover analysis), and *Z*-test were performed. Repeated-measures analysis was used to test the carryover effect and period effect. For statistical significance, *p* value less than 0.05 was used. Statistical analysis was done by the Statistical Package for Social Science PC+, version 19.0 (SPSS Inc., Chicago, IL, USA).

## 3. Results

### 3.1. Study Population

Of a total of 41 subjects, 9 subjects were excluded, and 32 subjects were randomly divided into KRG group and placebo group. One subject wanted to quit due to side effect (gastric discomfort), and 8 subjects dropped out during the study because of a lack of subjective improvement. Finally, 23 subjects completed the study (Table 1). Twelve subjects started KRG medication first and placebo medication later (KRG-placebo group). Eleven subjects started placebo medication first and KRG medication later (placebo-KRG group). All of them were without other medical history except only one participant who had combined hypothyroidism.

### 3.2. Sexual Function: FSFI

Total FSFI scores increased after KRG treatment (from 20.13 ± 2.87 to 23.98 ± 4.10, *p* = 0.015) and placebo treatment (from 20.06 ± 2.64 to 23.78 ± 3.28, *p* = 0.003). However, this change was not significantly different compared to that of placebo group (*p* = 0.702). No period effect or carryover effect was observed. The sexual desire was significantly improved after 8-week treatment of KRG while there was no significant effect in placebo. However, there was no significant treatment effect between two groups. Overall, KRG treatment significantly improved sexual desire, arousal, orgasm, and satisfaction domains; however, there was no treatment effect compared with placebo (Table 2).

### 3.3. Hormone Levels and Side Effects

Changes in sexual hormones during the study were described in Table 3. Mean total testosterone level increased from 0.24 ± 0.89 ng/mL to 0.29 ± 0.12 ng/mL and estradiol increased from 153.99 ± 88.93 pg/mL to 175.60 ± 149.77 pg/mL in the KRG group. However, similar findings were also observed in the placebo group. There were no statistically significant changes between KRG treatment and placebo group (testosterone, *p* = 0.618; estradiol, *p* = 0.450).

Only one adverse event was reported in this study. It was a mild gastric discomfort after intake of KRG.

## 4. Discussion

Female sexual dysfunction may worsen the whole life of affected women [22, 23]. Women with sexual dysfunction showed lower quality of life and less satisfaction with their partner [22]. Female sexual dysfunction was associated with psychological and emotional distress as well as significantly lower sexual satisfaction, lower self-esteem, and more anxiety [24, 25].

Among the complementary and alternative medicines with improving sexual function, ginseng appears to be one of the attractive options. Ginseng has several pharmacological activities [26, 27]. In an animal model study, we found that KRG relaxed the clitoral corpus cavernosal smooth muscle and vaginal smooth muscle in rabbits by the NO-cGMP pathway. Also, KRG hyperpolarized the Ca^2^-activated K^+^ channels [18]. Ginseng enhances the NO synthesis in the endothelium and acts as an antioxidant with a protective effect [13, 28]. These effects of ginseng have potential improvement of female sexual function. In menopausal women, we reported that improvement of female sexual function (particularly arousal) was shown after 8 weeks' intake of KRG [12]. According to previous study, we hypothesized that KRG may improve sexual function in premenopausal women.

In KRG treatment group of this study, FSFI scores were significantly increased after medication. However, improvement of FSFI scores in the KRG group was not statistically different compared to placebo group. There were possible explanations as follows. First, there was a large placebo response which makes a difficulty in demonstrating a difference between active treatment and placebo groups. Bradford reported that 40% or more of women improved significantly after a placebo intervention for hypoactive sexual desire disorder and female sexual arousal disorder [8]. Second, there may be an influence of menstrual cycle of the subjects. We did not consider the menstrual cycle of the subject when we checked a questionnaire. Roney and Simmons reported that hormone level was associated with sexual behavior [29]. According to their study, women's subjective sexual desire exhibited in periovulatory peaks and estrogen had a positive effect on sexual desire. For a more accurate assessment, interviewing the subjects of study at similar point of menstrual cycle is needed.

We previously reported the KRG improved sexual function in menopausal women [12]. After 8 weeks of treatment of KRG, there was an improvement of sexual function in sexual arousal domain. It may be associated with a phytoestrogen effect of ginseng [19]. There was a report that red ginseng could relieve menopausal symptoms [30].

Importantly, KRG did not show significant side effect compared to placebo in our study. There was one adverse event of a mild gastric discomfort after KRG treatment. KRG was a traditional herbal medicine that has been used for maintenance and improvement of human health in Asian countries for a long time. In the laboratory test, there were no significant findings compared to placebo.

The present study had some limitations. First, we did not check the partners' sexual function. Female sexual function may be influenced by partner's sexual status. Second, there was no study to prove an optimal dose of KRG for improving sexual function. Third, we did not consider a menstrual cycle of participants in this study. Menstrual cycle may influence a hormone level in women.

## 5. Conclusion

Oral administration of KRG extracts improved sexual function in premenopausal women; however, there were no statistical significant changes compared to placebo. It implies that KRG extracts have a substantial placebo effect in premenopausal women with sexual dysfunction. Further studies are needed to evaluate the effect of KRG extracts on specific disorders of female sexual dysfunction, such as female sexual desire disorder or arousal disorder.

## Figures and Tables

**Table 1:** Baseline characteristics of patients.

	KRG-placebo (*n* = 12)	Placebo-KRG (*n* = 11)	*p* value
Age (yr)	38.25 ± 5.92	37.36 ± 6.28	0.449
Marriage duration (yr)	12.92 ± 8.26	12.09 ± 7.30	0.651
Duration of FSD	2.88 ± 1.69	2.05 ± 1.17	0.608
Chronic medical disease, *n*			
Hypothyroidism	0	1	
Delivery history, *n*			
Vaginal	9	8	
Cesarean section	3	3	
FSFI	21.53 ± 3.19	20.12 ± 3.58	0.316

Data are presented as mean ± standard deviation unless otherwise indicated.

KRG: Korean red ginseng; FSD: female sexual dysfunction; FSFI: Female Sexual Dysfunction Index.

**Table 2:** The results of Female Sexual Function Index in the Korean red ginseng and placebo groups.

	KRG (*n* = 23)			Placebo (*n* = 23)			Period effect * *(*p*)	Carryover effect * * (*p*)	Treatment effect * * (*p*)^‡^
	Baseline	8 wk	*p* ^†^	Baseline	8 wk	*p* ^†^			
Desire	2.70 ± 0.65	3.70 ± 0.80	0.003	2.50 ± 0.64	3.05 ± 0.73	0.058	0.514	0.947	0.228
Arousal	3.00 ± 0.72	3.72 ± 0.90	0.040	2.92 ± 0.55	3.55 ± 0.81	0.017	0.693	0.883	0.431
Lubrication	3.97 ± 0.78	4.23 ± 0.84	0.562	3.76 ± 0.81	4.31 ± 0.80	0.054	0.022	0.306	0.770
Orgasm	3.20 ± 0.64	3.90 ± 1.01	0.028	3.42 ± 0.60	4.18 ± 0.62	0.007	0.288	0.789	0.288
Satisfaction	3.40 ± 0.50	4.00 ± 0.85	0.027	3.49 ± 0.60	3.89 ± 0.69	0.040	0.354	0.579	0.894
Pain	3.87 ± 1.17	4.43 ± 1.34	0.212	3.96 ± 1.30	4.80 ± 0.74	0.012	0.764	0.411	0.096

Total	20.13 ± 2.87	23.98 ± 4.10	0.015	20.06 ± 2.64	23.78 ± 3.28	0.003	0.266	0.535	0.702

KRG: Korean red ginseng.

^†^Wilcoxon signed rank test using baseline and treatment of 8 weeks.

^‡^Wilcoxon signed rank test using KRG and placebo.

**Table 3:** Changes of hormone level after each medication.

		KRG group (*n* = 23)	Placebo group (*n* = 23)	Period effect (*p*)	Carryover effect (*p*)	Treatment effect (*p*)
Total testosterone (ng/mL)	Baseline	0.24 ± 0.19	0.24 ± 0.14	0.310	0.510	0.618
	8 wk	0.29 ± 0.12	0.29 ± 0.18			

Free testosterone (ng/mL)	Baseline	0.49 ± 0.23	0.49 ± 0.25	0.426	0.483	0.628
	8 wk	0.49 ± 0.24	0.52 ± 0.31			

Estradiol (pg/mL)	Baseline	153.99 ± 88.93	124.46 ± 87.99	0.231	0.612	0.450
	8 wk	175.60 ± 149.77	150.13 ± 140.30			
